# Ozone-Assisted Green Upgrading of *Lactuca sativa* Oil: Characterization and Bioactivity for Clean-Label Functional Applications

**DOI:** 10.3390/foods14203458

**Published:** 2025-10-10

**Authors:** Abdulrahman S. Bazaid, Sulaiman A. Alsalamah, Waleed Hakami, Mohammed Ibrahim Alghonaim, Amro Duhduh, Husam Qanash

**Affiliations:** 1 Department of Medical Laboratory Science, College of Applied Medical Sciences, University of Ha’il, Hail 55476, Saudi Arabia; h.qanash@uoh.edu.sa; 2 Department of Biology, College of Science, Imam Mohammad Ibn Saud Islamic University (IMSIU), Riyadh 11623, Saudi Arabia; saalsalamah@imamu.edu.sa (S.A.A.); mialghonaim@imamu.edu.sa (M.I.A.); 3 Department of Medical Laboratory Technology, College of Nursing and Health Sciences, Jazan University, Jazan 45142, Saudi Arabia; wmhakami@jazanu.edu.sa (W.H.); aduhduh@jazanu.edu.sa (A.D.); 4 Medical and Diagnostic Research Center, University of Ha’il, Hail 55473, Saudi Arabia

**Keywords:** lettuce oil, ozonation, *Helicobacter pylori*, butyrylcholinesterase inhibition, antioxidant, antihemolytic

## Abstract

Ozonation is an emergent green technology that modifies the chemical composition and bioactivity of natural oils, creating new opportunities for functional and biomedical use. In this study, the chemical changes and in vitro activities of lettuce (*Lactuca sativa*) oil before and after ozonation were evaluated. Gas chromatography–mass spectrometry (GC–MS) revealed an increase in both the number and diversity of constituents in ozonated oil, with (Z)-13-docosenamide and trans-13-octadecenoic acid as predominant components. Fourier-transform infrared (FTIR) spectra showed overall similarity between native and ozonated oils, but with three additional characteristic bands in the ozonated sample. Bioassays demonstrated that ozonation enhanced anti-*Helicobacter pylori* activity (inhibition zone 21.3 ± 0.3 mm), supported bactericidal effects, and improved antibiofilm and antihemolytic properties. The antioxidant capacity of ozonated oil was modestly increased (IC_50_ = 3.95 ± 0.4 µg/mL), while butyrylcholinesterase inhibition was more markedly enhanced (IC_50_ = 2.58 ± 0.6 µg/mL), compared to that of the non-ozonated oil (IC_50_ = 6.14 ± 0.3 µg/mL and IC_50_ = 4.38 ± 0.4 µg/mL, respectively). Molecular docking suggested strong interactions of major ozonation-derived compounds with human BuChE and *H. pylori* urease, providing mechanistic support for the observed activities. Overall, these results indicate that ozonation modestly but consistently enhances the biological potential of lettuce oil through compositional shifts, highlighting its promise for development as a safe functional food ingredient with possible biomedical applications.

## 1. Introduction

Most of the world’s botanical species offer immense potential for research and for the development and manufacture of novel therapeutics that benefit humanity. Numerous contemporary strategies are available to discover new biologically active plant-derived substances suitable for the production of safe medications [1,2,3]. Extensive scientific efforts have focused on analyzing and identifying antioxidants, antimicrobial, and antifungal compounds from diverse natural sources, including soil, microbes, animals, and plants. Traditional botanical remedies, in their various forms, remain among the most valuable resources in this endeavor [4,5]. Systematic evaluation of these conventional plants can yield previously unrecognized bioactive constituents for functional food development. In particular, essential oils and crude botanical extracts attract interest because of their favorable safety profiles and widespread consumer use; these preparations contain natural chemicals that frequently exhibit multipurpose activities, such as antibacterial and antioxidant effects [6,7].

*L. sativa*, commonly known as lettuce, is a member of the Asteraceae family and is widely cultivated as a leafy vegetable [8,9]. First domesticated by the ancient Egyptians, it later spread to Europe and other regions. Lettuce is now cultivated in tropical and subtropical areas and provides essential nutrients, including vitamins and minerals, important for human health [10,11].

Ozonation of organic oils involves reacting medical-grade ozone with the unsaturated fatty acids present in oils (e.g., olive oil) to generate ozonides and peroxides, which confer biological activity [12,13]. The process is governed by ozone concentration, flow rate, and oil type, yielding stable oxygenated molecules that gradually release ozone and have applications in medicine and aesthetics. The presence of water during ozonation promotes peroxide formation, potentially enhancing antibacterial properties. Oils with a higher degree of unsaturation react more rapidly with ozone [14,15,16]. Lettuce oil is an underutilized yet nutritionally valuable edible oil, characterized by a high content of polyunsaturated fatty acids, primarily linoleic and oleic acids, along with tocopherols, phytosterols, and phenolic compounds [8,11]. These bioactive constituents not only provide inherent antioxidant and health-promoting effects but also serve as reactive precursors for the formation of oxygenated derivatives upon ozonation. Compared with other oils commonly investigated for ozonation, such as black seed and pumpkin seed oils [12,13], lettuce oil offers distinct advantages. Its elevated degree of unsaturation makes it particularly susceptible to ozone attack, thereby facilitating the generation of a broader range of ozonides and peroxides with potentially enhanced antimicrobial and antioxidant activities.

*H. pylori* is a bacterium that causes gastritis and peptic ulcers of the gastrointestinal tract; infections may produce symptoms such as diarrhea, abdominal pain, and nausea or remain asymptomatic [17,18]. Standard *H. pylori* therapy typically includes at least two antibiotics and a proton-pump inhibitor (PPI) to control gastric acid and in some cases bismuth subsalicylate to protect the gastric lining [19]. Important limitations of current regimens include rising antibiotic resistance, the complexity of taking multiple drugs, and the risk of reinfection after therapy [20,21].

Docking studies of natural compounds employ computational techniques to predict how small molecules interact with biological targets, thereby aiding the identification of candidate drugs, elucidating potential mechanisms of action, and reducing the time and cost of discovery [22]. Typical workflows involve preparing protein structures and natural-product ligands, performing docking calculations to determine optimal binding poses, and analyzing scores that estimate binding affinity and key interactions, such as hydrogen bonds and hydrophobic contacts. This approach accelerates natural-product screening by rapidly pinpointing prospective bioactive phytochemicals from large databases [23].

Despite the wide use of plant-derived oils as natural therapeutics, limited information is available regarding the impact of ozonation on the chemical composition and biological activities of lettuce oil, particularly against *H. pylori* and in relation to neuroprotective potential. Unlike common oils that have been extensively studied, lettuce oil has received limited attention, particularly with respect to its potential in controlling *H. pylori* and Alzheimer’s disease. Furthermore, its functional enhancement through eco-friendly processing such as ozonation remains poorly documented. By focusing on this unconventional oil, our study introduces novelty, expands the knowledge of underexplored edible oils, and opens opportunities for developing niche, clean-label functional ingredients. Therefore, the present study specifically selects lettuce oil with the aim of characterizing its chemical profile before and after ozone exposure using GC–MS and FTIR analyses, while also evaluating the antibacterial, antibiofilm, and antihemolytic properties of ozonated and non-ozonated lettuce oils against *H. pylori*, as well as assessing their antioxidant and butyrylcholinesterase inhibition potential.

## 2. Materials and Methods

### 2.1. Chemicals and Ozonation Process

All chemicals and reagents were obtained from Sigma (Cairo, Egypt). Lettuce oil was sourced from a specialized, authenticated company (Code: 03652). The oil was extracted from seeds by cold pressing and used in its virgin (unrefined) form. The peroxide value was determined to be 3.8 meq O_2_/kg oil, and the p-anisidine value was 2.6. Ozone gas was generated using an electric boundary shockwave plasma generator. The plasma reactor outlet was connected to a chilling bath set at −9 °C and submerged in a 1.7 L Drechsel cylinder containing 1.0 L of lettuce oil. Ozone was bubbled through the oil for six hours at an average flow rate of 0–8 L min^−1^, during which the oil assumed a semisolid consistency. The ozonated oil was collected from the Drechsel vessel, transferred to a clean, empty glass container, and stored at 8 °C [24,25].

### 2.2. GC–MS Analysis

For analysis of the lettuce oil types, gas chromatography (Thermo Scientific, ISQ 7000 Quadrupole GC–MS, Waters, Milford, MA, USA) coupled to an ISQ Dual Quadrupole mass analyzer (GC–MS) was used with high entitativity detector (MS). Separations were performed on a TR-5MS capillary column (29 m × 0.35 mm i.d., 0.257 µm film). The oven program was initial 61 °C, ramp to 242 °C, then increase at 31 °C min^−1^ to 292 °C, with a 2.0 min isothermal hold. The injector temperature was 252 °C, and the MS transfer line was maintained at 265 °C. Helium (highest grade) was employed as the carrier gas at a constant flow of 1.0 mL min^−1^. One microliter of each test oil was injected in split mode using an autosampler (AS1301) connected to the GC. Electron-ionization spectra were acquired in full-scan mode over m/z 40–1000 at 70 eV. Components were identified by retention time (RT) and mass spectral matching against the National Institute of Standards and Technology (NIST) library [26].

### 2.3. FTIR Analysis

Oil samples were analyzed at ambient temperature after forming a thin film on a metallic mesh. Spectra were collected using a Bruker VERTEX 70 series FTIR spectrometer (Bruker Optics, Ettlingen, Germany) and corrected using the mesh-cell background. The optical path of the oil samples was standardized to 0.15 mm, and spectra were recorded over 400–3549 cm^−1^. Data processing was performed with OMNIC 7.3 and TQ Analyzer 7.2 software [27].

### 2.4. Anti-H. pylori Assay

The antibacterial was assessed using *H. pylori* (ATCC 26695). In vitro anti-*H. pylori* activity was determined by a standard agar diffusion (well) method. Briefly, 100 µL of a bacterial suspension (10^8^ CFU/mL) was spread on Mueller–Hinton agar supplemented with 10% blood and blood products. After solidification, 6 mm wells were bored using a sterile cork borer, and test samples were added [28]. Clarithromycin was used as the standard antibiotic (positive control) in the anti-*H. pylori* assay at a concentration of 10 µg/mL.

### 2.5. Minimum Inhibitory Concentration (MIC) and Minimum Bactericidal Concentration (MBC)

MICs were determined by microbroth dilution in Mueller–Hinton broth against *H. pylori*. The tested oils were prepared in a dilution series ranging from 0.98 to 1000 µg/mL. An aliquot (200 µL) of each dilution was dispensed into 96-well plates. A fresh *H. pylori* inoculum was adjusted in sterile NaCl (0.86%) to McFarland 1.0. Then, 2.0 µL of the inoculum was added to each well to achieve a final concentration of 5 × 10^3^ CFU/mL. Plates were incubated at 37 °C for 72 h. MICs were visually recorded as the lowest concentrations that inhibited visible growth. Negative controls contained the tested oils without bacteria, and positive controls contained the *H. pylori* inoculum without oils were included [29].

MBCs were determined by taking 10 µL from wells showing no visible growth and spotted onto Mueller–Hinton agar. Plated were incubated at 37 °C for 72 h. The MBC was defined as the lowest concentration that completely prevented H. pylori growth on agar. The MBC/MIC ratio was used to classify activity; ratios < 4 were interpreted as bactericidal [30].

### 2.6. Anti-Biofilm Activity

Biofilm-formation inhibition was assessed in 96-well microplates. Each well contained 280 µL of freshly inoculated trypticase soy–yeast (TSY) broth, standardized to a final density of 10^6^ CFU/mL. Microplates were then incubated with test oils at previously established sublethal doses corresponding to 75%, 50%, and 25% of the MBC. Wells containing medium only, vehicle (alcohol) or specimen served as controls. Plates were incubated at 37 °C for 48 h. Supernatants were removed and wells were gently rinsed with sterile distilled water to remove planktonic cells. After air-drying for 30 min, adherent biofilms were stained with 0.11% crystal violet for 15 min at room temperature. Excess stains were removed with three washes of sterile distilled water. Bound dye was then solubilized with 250 µL of 96% ethanol, and absorbance was read at 575 nm using a microplate reader [25].

### 2.7. Anti-Hemolytic Assay

Anti-hemolytic efficacy was evaluated at sub-MIC levels of 25%, 50% and 75% in *H. pylori*-exposed conditions. Following treatment, cultures were adjusted to OD_600_ = 0.40 and centrifuged at 20,000× *g* for 25 min. Supernatants (500 µL) were mixed with 0.8 mL saline containing a 2% erythrocyte suspension, incubated for 2 h at 37 °C, and centrifuged for 10 min at 12,000× *g* and 6 °C. Negative controls (NCs) comprised un-hemolyzed erythrocytes incubated in Luria–Bertani (LB) broth; positive controls (PCs) were prepared by adding 0.1% sodium dodecyl sulfate (SDS) to the erythrocyte mixture. Hemoglobin release was quantified by absorbance at 545 nm. For sub-MIC–treated bacteria, results were reported as the percent change in oil-induced hemolysis relative to untreated reference cultures [31].

### 2.8. Antioxidant Assay

Antioxidant capacity was measured using the 2,2-diphenyl-1-picrylhydrazyl (DPPH) radical-scavenging assay. Forms of lettuce oil were dissolved in DMSO, diluted to 1.9–1000 µg/mL, and mixed with 2 mL of 0.1 mM DPPH in methanol. After vortexing, mixtures were kept in the dark for 1 h. Negative control was prepared by combining 2 mL of DPPH solution with 1 mL methanol. Absorbance was recorded at 516 nm, and values represent the average of three independent measurements [32].

### 2.9. Butyrylcholinesterase Inhibition Assay

Butyrylcholinesterase (BChE) inactivation was assessed using a modified Ellman procedure. Fresh buffer and BChE solutions were prepared. An S-butyrylthiocholine iodide (BTChI) solution (0.02 M; 6.9 mg BTChI in 1.0 mL water) and a BChE solution (0.45 U mL^−1^; 2.9 mg BChE enzyme in 6.7 mL buffer, pH 7.9) were used. Each specimen was first dissolved in DMSO and then in distilled water to 44 mg/ mL, yielding a final test concentration of 1000 µg/mL. For each assay, 200.0 µL buffer, 5 µL BChE enzyme, 5.0 µL Ellman’s reagent [5,5′-dithiobis (2-nitrobenzoic acid), DTNB], and 5 µL of the specimen at 40 mg mL^−1^ were combined and incubated in a temperature-controlled water bath for 15 min at 30 °C. The reaction was initiated by adding 5 µL of the BTChI substrate solution. Absorbance at 420 nm was measured 13 times at 44 s intervals using a microplate reader maintained at 35 °C [33].

### 2.10. Molecular Docking Studies

Molecular docking was performed using Molecular Operating Environment (MOE) software version 2019 (Chemical Computing Group Inc., Montreal, QC, Canada) to evaluate binding interactions between the ligands [13-Docosenamide, (Z) and trans-13-Octadecenoic acid] and target proteins [34]. Crystal structures of human butyrylcholinesterase (BChE, PDB ID: 4TPK) and *H. pylori* urease (PDB ID: 1E9Z) were retrieved from the Protein Data Bank. Protein preparation included addition of hydrogen atoms, removal of water molecules, and energy minimization. Ligand structures were constructed and energy-minimized within MOE. Active binding sites were identified using MOE Site Finder and defined as dummy atoms for docking; the pocket was retained in MOE for predicting ligand–protein interactions. Ligands were docked into the defined sites using Triangle Matcher placement followed by Rigid Receptor refinement in MOE [35,36]. Poses were initially scored with the London dG function and rescored with GBVI/WSA dG. Multiple docking runs were conducted for each ligand to obtain the best binding conformations. Docking outcomes were evaluated using docking score (S), rmsd_refine, and energy terms (E_conf, E_place, E_score1, E_refine, E_score2). Interaction analyses were performed with MOE tools to identify hydrogen bonds, hydrophobic interactions, and π-interactions. Two-dimensional and three-dimensional interaction diagrams were generated to visualize ligand binding within the active sites of the target proteins [37].

### 2.11. Statistical Analysis

All tests were performed in triplicate. Data are presented as the mean ± SD. Differences between two groups were analyzed using the t-test, and studies involving multiple variances were analyzed by one-way ANOVA, both conducted in GraphPad Prism v8 (GraphPad Software, San Diego, CA, USA). Statistical significance was accepted at *p* < 0.05.

## 3. Results and Discussion

### 3.1. GC–MS Chemical Profile and Ozonation-Induced Shifts

**Figure 1:**
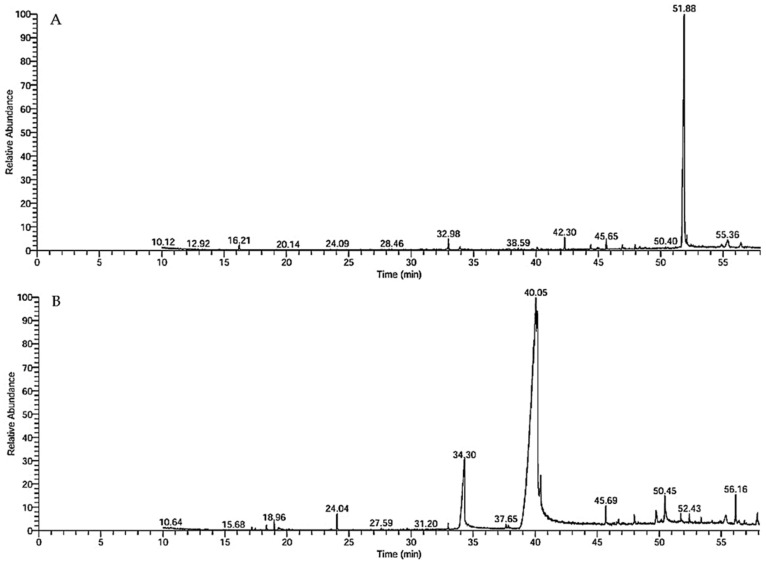
GC–MS chromatograms of (**A**) crude lettuce oil and (**B**) ozonated lettuce oil.

**Figure 2:**
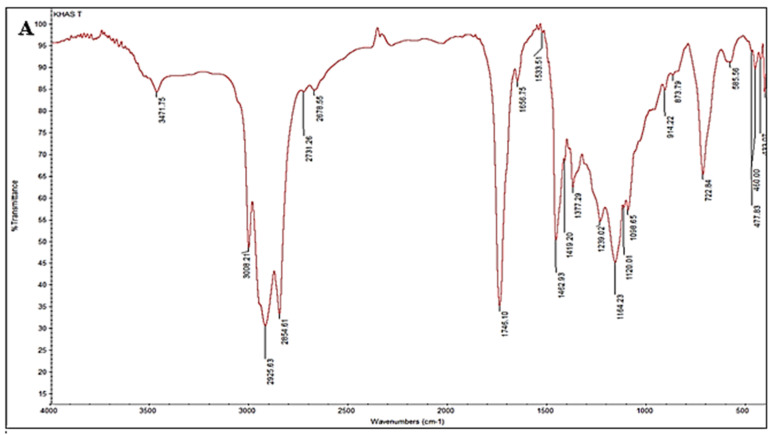

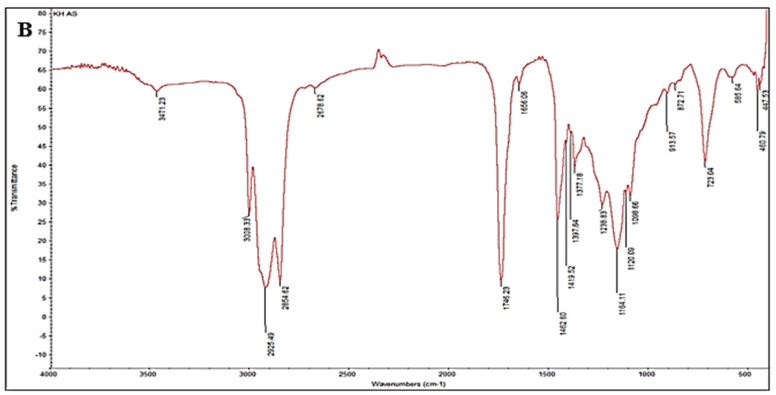
FTIR spectra of (**A**) crude lettuce oil and (**B**) ozonated lettuce oil.

The various chemical constituents of lettuce oils were readily detected by GC–MS (Table 1; Figure 1 and Figure 2). The raw lettuce oil contained 16 molecules spanning 10 chemical classes, whereas the ozonated oil contained 22 molecules across 12 classes. The predominant compounds present in both oils were (Z)-13-docosenamide and trans-13-octadecenoic acid, with notable differences in their relative abundances. Six additional constituents were shared by both oil forms: 7,9-di-tert-butyl-1-oxaspiro(4,5)deca-6,9-diene-2,8-dione (spirocyclic diketone); trans-13-octadecenoic acid (fatty acid); phenol, 2,2′-methylenebis[6-(1,1-dimethylethyl)-4-methyl-] (phenol); (Z)-13-docosenamide (fatty amide); ethyl iso-allocholate (steroid); and dotriacontane (alkane). Mechanistically, ozonation converts unsaturated fatty acids into oxygenated derivatives, including aldehydes, carboxylic acids, and ozonides, thereby reshaping the chemical profile and effectively decreasing overall unsaturation; consistent with this, ozonated oils typically show increased proportions of decomposition products, with several carboxylic acids and aldehydes (notably hexanal and nonanal) becoming prominent constituents of the chemical fraction [38,39]. Collectively, these compositional shifts highlight ozonation as a precise, green modification strategy that reprograms lettuce oil chemistry toward bioactive oxygenated species, an effect that dovetails with, and helps rationalize, the enhanced biological activities reported in this study.

### 3.2. FTIR Spectral Signatures and Ozonation Effects

The FTIR pattern of native lettuce oil exhibited 23 characteristic bands at 3471.75, 3008.21, 2925.63, 2854.61, 2731.26, 2676.55, 1746.10, 1656.75, 1533.51, 1462.93, 1419.20, 1377.29, 1239.02, 1164.23, 1120.01, 1088.65, 914.22, 873.79, 722.84, 585.56, 477.83, 460.00, and 433.07 cm^−1^ (Figure 2A). In this spectrum, the 3500–3000 cm^−1^ region is taken to indicate features associated with unsaturation (C=C), the 1650–2000 cm^−1^ region is attributed to C–H bending, and the sharp band at 1746 cm^−1^ corresponds to C=O stretching of carbonyl groups in triacylglycerols, the principal constituents of vegetable oils. The 400–1650 cm^−1^ region is assigned to C–O and C–C skeletal vibrations in lettuce oil. Overall, FTIR shows promising utility for the classification of natural oils [40].

By comparison, the FTIR pattern of ozonated lettuce oil displayed 20 bands in analogous regions at 3471.23, 3008.31, 2925.49, 2854.62, 2676.62, 1746.23, 1656.06, 1462.60, 1419.52, 1377.18, 1238.83, 1164.11, 1120.09, 1096.66, 913.57, 872.71, 723.04, 585.64, 460.79, and 447.53 cm^−1^ (Figure 2B). Notably, the bands at 2731.26, 1533.51, and 477.83 cm^−1^ observed in the native oil were absent after ozonation. Mechanistically, ozonation disrupts double bonds in unsaturated fatty acids, reflected here by loss of C=C features (around 1500 cm^−1^) and the appearance of bands attributable to ozonides [41]. In addition, the band near 2700 cm^−1^ in oil FTIR spectra, typically associated with C–H stretching in aldehydes, was not evident post-ozonation, consistent with a decrease in aldehyde functional groups due to oxidative transformation (e.g., oxidation or epoxidation) into other functionalities [42]. Furthermore, the disappearance of a band near 400 cm^−1^ suggests the loss of a specific molecular feature, likely a chiral group, during the chemical process, a phenomenon used to monitor oxidative changes in oil composition [43].

Together, these spectral shifts furnish a coherent molecular fingerprint of ozonation in lettuce oil, reinforcing the compositional reprogramming toward oxygenated species that underpins the enhanced bioactivities documented in this study.

### 3.3. Anti-H. pylori Activity and Bactericidal Efficacy

**Figure 3:**
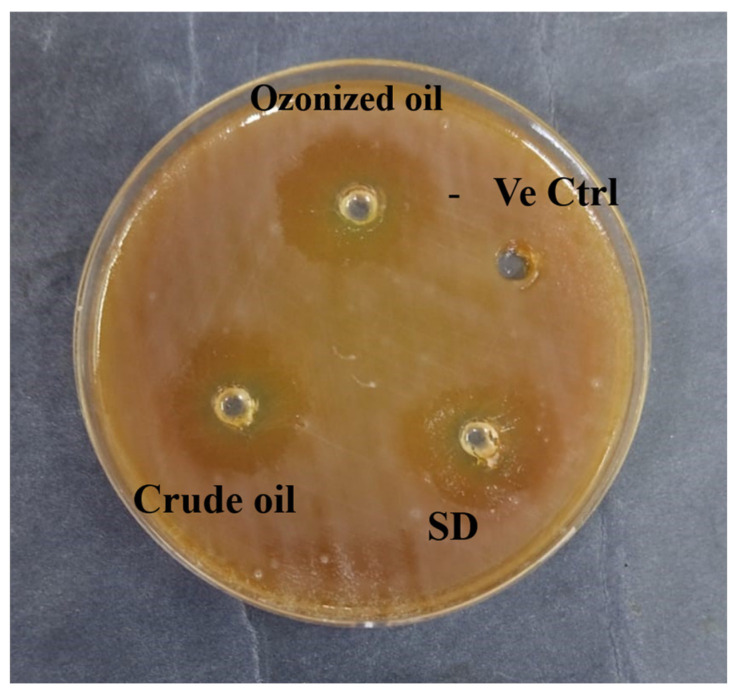
Anti-*H. pylori* activity of crude and ozonated lettuce oils assessed by the agar well-diffusion assay.

In this work, native lettuce oil exhibited appreciable anti-*H. pylori* activity with an inhibition zone of 13.7 ± 0.6 mm, compared with the standard drug (15.3 ± 0.4 mm). By contrast, ozonated lettuce oil produced a larger inhibition zone of 21.3 ± 0.3 mm. These findings are corroborated by minimum inhibitory concentration (MIC) and minimum bactericidal concentration (MBC) analyses, where ozonated lettuce oil displayed significantly lower MIC values (31.25 ± 0.5 µg/mL) and MBC values (62.5 ± 0.3 and 125.5 ± 0.8 µg/mL) compared with the native oil and both oil forms demonstrated bactericidal action against *H. pylori* (Figure 3; Table 2). The superior efficacy of ozonated lettuce oil can be attributed to its enriched content of oxygenated derivatives that arise from ozonolysis of unsaturated fatty acids which have been shown to disrupt bacterial cell membranes, interfere with protein functions, and induce oxidative stress within microbial cells [24]. In the case of *H. pylori*, which colonizes the gastric mucosa by producing urease and adapting to oxidative stress, these oxygen-enriched metabolites may synergistically impair bacterial survival mechanisms, thereby explaining the observed reduction in MIC and MBC values.

The present findings align with prior studies demonstrating the antimicrobial activity of oils following ozonation. For example, ozonated sunflower and olive oils have been reported to exhibit enhanced antibacterial effects against Gram-positive and Gram-negative pathogens [38]. Ozonated pumpkin seed oil was recently shown to produce larger inhibition zones and superior bactericidal action compared with its native counterpart (Alsalamah et al., 2025) [13]. This suggests that ozonation represents a broadly applicable green biotransformation strategy for augmenting the antimicrobial potential of plant-derived oils.

The therapeutic management of *H. pylori* infections is becoming increasingly challenging due to the global surge in resistance to antibiotics [44]. This growing resistance crisis necessitates the exploration of novel, cost-effective, and sustainable alternatives. Ozonated lettuce oil is a compelling candidate, capable of exerting potent bactericidal activity at relatively low concentrations. Its natural origin, favorable safety profile, and eco-friendly production process further strengthen its appeal for translational evaluation.

### 3.4. Antibiofilm Activity Against H. pylori

**Figure 4:**
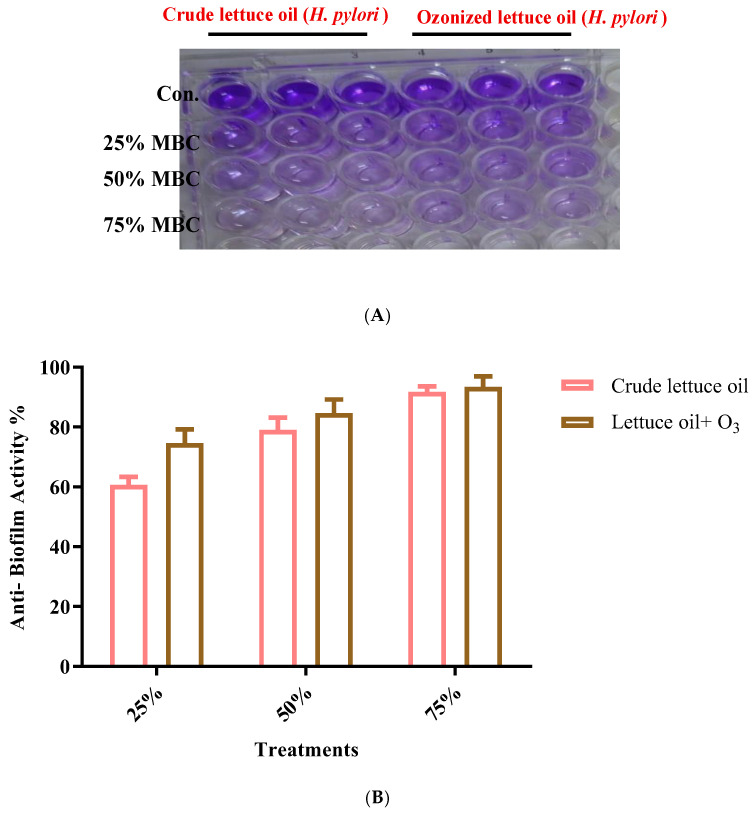
Antibiofilm activity against *H. pylori* of crude and ozonated lettuce oils. (**A**) Representative 96-well plate at various percentages of the MBC. (**B**) Bar graphs depicting differences between the oil forms. “Con.” in panel A denotes the control.

Exposure of lettuce oil to ozone produced a slight improvement in antibiofilm activity against *H. pylori*, with no statistically significant differences (*p* > 0.05) across the tested sublethal concentrations corresponding to 25–75% of the MBC (Figure 4). While the magnitude of improvement was modest, the consistent directional aligned with the broader evidence suggesting that ozonation of vegetable oils can subtly modulate antibiofilm properties [45]. However, several vegetable oils possess intrinsic antibiofilm effects, disruption of bacterial cell-to-cell communication (quorum sensing), impeding biofilm formation and facilitating biofilm removal thereby offering complementary or alternative options to conventional antibiotics [46]. Literature further indicates that ozonation can augment these effects, with enhanced antibiofilm performance contributing to the overall anti-*H. pylori* efficacy of ozonated oils [14,45]. Although lettuce oil showed mild antibiofilm activity, the ability of ozonated lettuce oil to act simultaneously as a bactericidal agent and a biofilm-disrupting adjunct positions it as a biofilm-conscious therapeutic candidate. Integration of ozonated oils with standard eradication regimens may improve treatment efficacy by weakening biofilm resilience, thereby enhancing antibiotic penetration among *H. pylori* communities.

### 3.5. Antihemolytic Activity

**Figure 5:**
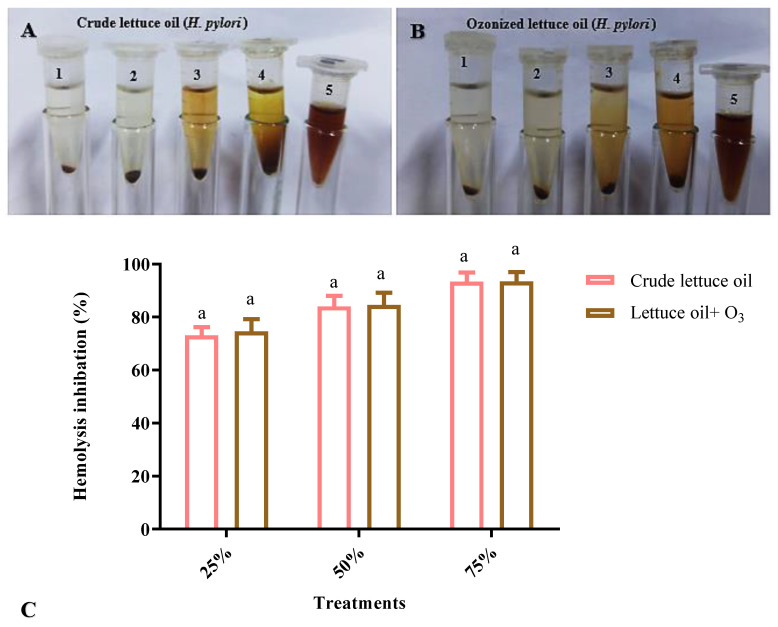
Inhibition of hemolysis in the presence of *H. pylori* by crude (**A**) and ozonated (**B**) lettuce oils. For both panels, conditions are arranged as follows: (1) *H. pylori* only, (2) 25% MIC, (3) 50% MIC, (4) 75% MIC, and (5) untreated; the SDS-induced complete-hemolysis control is also designated as 5 in the original panel. (**C**) Bar graphs illustrate differences between the oil forms; similar letters above columns indicate non-significant differences (*p* > 0.05).

Lettuce oil exhibited good antihemolytic activity against *H. pylori*–induced hemolysis, and ozonation produced a slight improvement in antihemolytic effects across various sub-MIC levels (Figure 5). Although direct prior evidence for antihemolytic properties of lettuce oil is limited, research indicates that essential oils from diverse plants can inhibit *H. pylori* by restraining bacterial growth, and certain lettuce constituents may likewise help counter the infection [47]. Additional studies are needed to evaluate specific lettuce types and to define their efficacy in managing *H. pylori*, particularly with respect to antihemolytic actions, despite promising activity reported for some oils and botanical extracts. Notably, altering the oil’s phytochemical profile (e.g., via ozonation) may enhance both suppression of *H. pylori* growth and stabilization of red blood cells, hallmarks of antihemolytic activity. In line with this rationale, ozonated oils (e.g., ozonated coconut oil) have shown enhanced effectiveness and promise as natural therapeutic options for infectious diseases, including viral illnesses [14,48]. Together, these observations nominate ozonated lettuce oil as an attractive, mechanism-informed adjunct for mitigating *H. pylori*–associated hemolysis and warrant focused mechanistic and in vivo evaluation.

### 3.6. Antioxidant Capacity (DPPH Assay)

**Figure 6:**
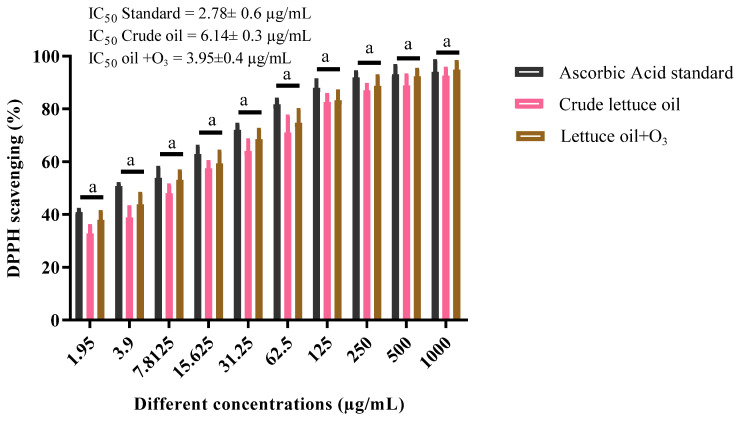
Antioxidant capacity of crude lettuce oil and ozonated lettuce oil relative to the ascorbic acid standard. Data are presented as the mean ± SD; similar letters above columns indicate non-significant differences (R^2^ value = 0.9859; *p* > 0.05).

In this work, crude lettuce oil exhibited appreciable antioxidant activity with an IC_50_ of 6.14 ± 0.3 µg/mL. Ozonation yielded a notable enhancement, reducing the IC_50_ to 3.95 ± 0.4 µg/mL, in contrast to ascorbic acid used as a reference standard (IC_50_ = 2.78 ± 0.6 µg/mL). There is a statistically significant difference (*p* ≤ 0.05) between the IC_50_ value of the ascorbic acid standard and the IC_50_ values for both crude and ozonized lettuce oils, with a modest increase in antioxidant capacity observed in the ozonized oil relative to the crude form (Figure 6). Reports indicate that ozonation can enhance the antioxidant properties of several oils [49]. This improvement is largely attributed to the formation of new antioxidant species, with the magnitude of the effect governed by the oil type, inherent composition, and the ozonation dose and duration [24,50]. Collectively, these findings show that controlled ozonation tunes the redox-active constituents of lettuce oil toward stronger radical scavenging, narrowing the gap with ascorbic acid and underscoring its promise as a green, bioactive antioxidant.

### 3.7. Butyrylcholinesterase Inhibition

**Figure 7:**
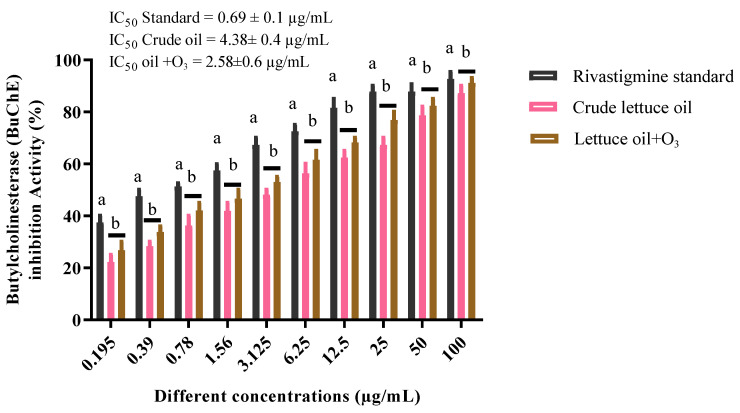
Butyrylcholinesterase (BChE) inhibition by crude lettuce oil and ozonated lettuce oil relative to the rivastigmine standard. Data are expressed as mean ± SD; different letters above the columns indicate significant differences among treatments (R^2^ value = 0.9555; *p* ≤ 0.05).

Native lettuce oil exhibited good butyrylcholinesterase (BChE) inhibitory activity with an IC_50_ of 4.38 ± 0.4 µg/mL. Upon ozonation, inhibition improved markedly, achieving an IC_50_ of 2.58 ± 0.6 µg/mL, compared with the rivastigmine reference standard (IC_50_ = 0.96 ± 0.6 µg/mL). There is a statistically significant difference (*p* ≤ 0.05) between the IC_50_ value of the rivastigmine standard and the IC_50_ values for both crude and ozonized lettuce oils, with a slight increase in inhibitory activity for the ozonized oil compared to the crude form (Figure 7). Cognitive dysfunction is a hallmark of Alzheimer’s disease, and BChE inhibition is a validated therapeutic strategy in its management. In support of this pharmacological axis, essential oils from *Citrus aurantifolia* and *Salvia* species contain constituents with strong BChE inhibitory potential [51]. Additionally, research indicates that specific fatty acids in vegetable oils can modulate BChE activity, aligning with the present observations. Consistent with previous reports, ozonation enhanced the butyrylcholinesterase inhibitory potential of natural oils, as evidenced by the stronger inhibition observed following ozonation. [48]. Taken together, these results show that ozonated lettuce oil narrows the gap to the reference inhibitor and merits targeted translational evaluation as a green, mechanism aligned BChE modulator.

### 3.8. Molecular Docking (BChE and H. pylori Urease)

The docking analyses showed that both ligands effectively engage the target proteins. For human BChE, 13-docosenamide displayed superior affinity (best docking score −8.13092 kcal/mol) relative to trans-13-octadecenoic acid (−7.31024 kcal/mol) (Table 3). Interaction analysis indicated that 13-docosenamide formed a hydrogen bond with GLU 276 (distance 3.42 Å, energy −0.6 kcal/mol) and an H–π interaction with TRP 82 (distance 3.72 Å, energy −0.7 kcal/mol) (Table 3). In contrast, trans-13-octadecenoic acid established a hydrogen bond with SER 287 (distance 2.97 Å, energy −1.3 kcal/mol) and an H–π interaction with TRP 82 (distance 3.69 Å, energy −0.8 kcal/mol) (Table 4).

**Figure 8:**
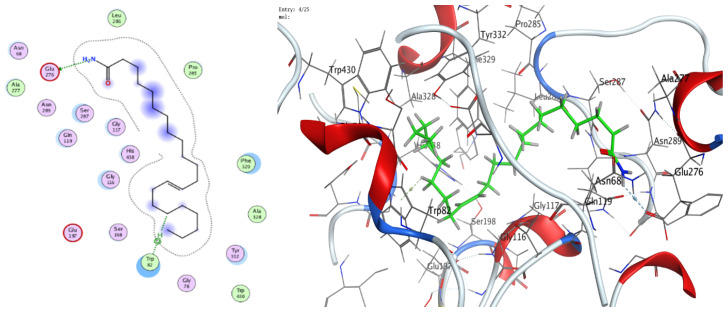
Two- and three-dimensional diagrams illustrating the interactions between (Z)-13-docosenamide and the active site of human butyrylcholinesterase (PDB: 4TPK).

**Figure 9:**
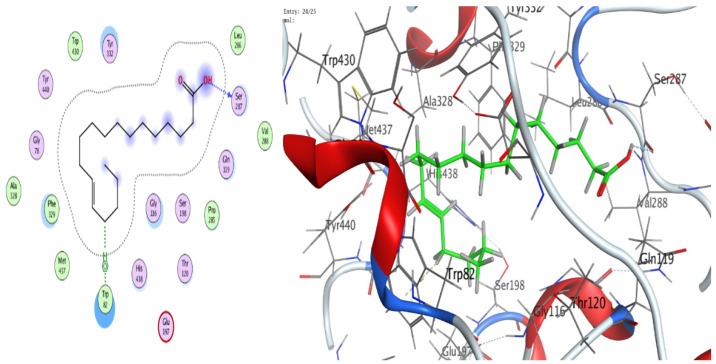
Two- and three-dimensional diagrams illustrating the interactions between trans-13-octadecenoic acid and the active site of human butyrylcholinesterase (PDB: 4TPK).

**Figure 10:**
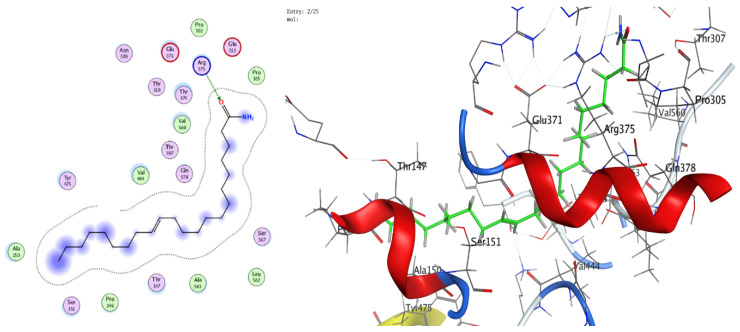
Two- and three-dimensional diagrams illustrating the interactions between (Z)-13-docosenamide and the active site of *H. pylori* urease (PDB: 1E9Z).

**Figure 11:**
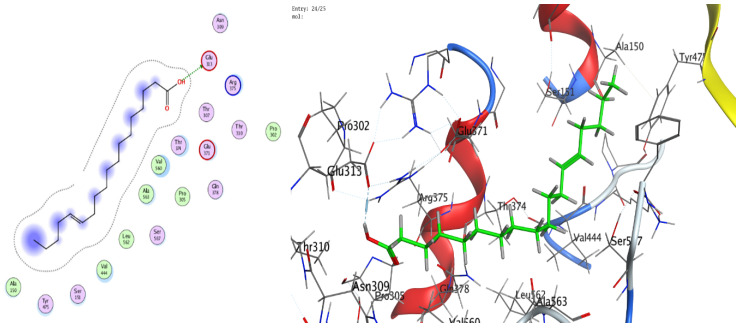
Two-dimensional (2D) and three-dimensional (3D) diagrams illustrate the interactions between trans-13-octadecenoic acid and the active sites of *H. pylori* urease (PDB: 1E9Z).

**Figure 12:**
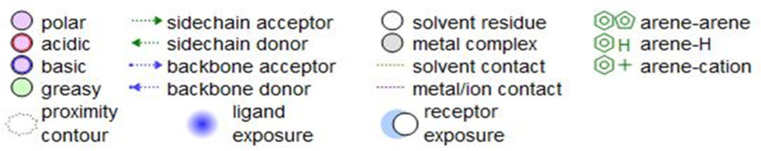
Representative key illustrating the interaction types between ligands and selected protein receptors.

For *H. pylori* urease, trans-13-octadecenoic acid exhibited stronger binding (best docking score −7.33015 kcal/mol) than 13-docosenamide (−6.00058 kcal/mol) (Table 5). The interaction profile showed a hydrogen bond between 13-docosenamide and ARG 375 (distance 3.05 Å, energy −0.7 kcal/mol) (Table 6), while trans-13-octadecenoic acid formed a particularly strong hydrogen bond with GLU 313 (distance 2.83 Å, energy −6.4 kcal/mol) (Table 5), likely contributing substantially to complex stability. Two-dimensional and three-dimensional interaction diagrams corroborated these binding modes within the active sites of both targets (Figure 8, Figure 9, Figure 10, Figure 11 and Figure 12).

Collectively, the docking results underscore 13-docosenamide as the more promising BChE binder, consistent with the enzyme’s relevance to neurological disorders and the therapeutic value of BChE inhibition in Alzheimer’s disease [52,53]. Conversely, trans-13-octadecenoic acid emerged as the stronger urease ligand, aligning with the enzyme’s pivotal role in *H. pylori* survival and pathogenicity and highlighting its potential as a lead for anti-*H. pylori* development [36,54]. These differences are readily rationalized by structural features: the amide functionality in 13-docosenamide supports distinct hydrogen-bonding patterns compared with the carboxylic acid group in trans-13-octadecenoic acid [55], while chain length and saturation modulate hydrophobic contacts and overall pose geometry [56]. Together, these in silico findings mirror the experimental bioactivity trends and nominate both molecules, via complementary target preferences, as tractable scaffolds for optimization and translational advancement.

## 4. Conclusions

Ozonation of lettuce oil, achieved by bubbling ozone at a flow rate of 0–8 L min^−1^, altered its chemical composition and generated new molecular classes. Compared with crude oil, the ozonated preparation exhibited higher in vitro anti-*H. pylori*, antibiofilm, antihemolytic, antioxidant, and butyrylcholinesterase inhibitory activities. Molecular docking supported these experimental findings and revealed complementary target preferences. (Z)-13-Docosenamide bound human butyrylcholinesterase with higher affinity, supporting its candidacy as an inhibitor relevant to neurological disorders including Alzheimer’s disease. Trans-13-octadecenoic acid displayed stronger binding to *H. pylori* urease, driven in part by a robust hydrogen bond with GLU313, indicating potential as an anti-*H. pylori* agent. Taken together, these results provide coherent in vitro evidence and a mechanistic foundation showing that ozonation transforms lettuce oil into a richer source of candidate therapeutics, and they motivate formulation, safety, and in vivo efficacy studies to advance translation. Future toxicology studies are needed to highlight the safety of ozonated lettuce oil using animal experiments.

## Figures and Tables

**Table 1:** Various chemical constituents identified in crude and ozonated lettuce oils by GC-MS.

Lettuce Oils						Ozonated Lettuce Oils					
Compound Name	Molecular Formula	Molecular Weight	RT (Raw)	Area (%)	Class	Compound Name	Molecular Formula	Molecular Weight	RT (O_3_)	Area (%)	Class
Benzaldehyde, 2,5-dimethyl-	C_9_H_10_O	134	16.21	0.97	Aromatic aldehyde	2-Decenal, (Z)-	C_10_H_18_O	154	17.45	0.16	Aldehyde
7,9-Di-tert-butyl-1-ox aspiro(4,5) deca-6,9-d iene-2,8-dione	C_17_H_24_O_3_	276	32 98	2.38	Spirocyclic diketone	2,4-Decadienal, (E, E)-	C_10_H_16_O	152	18.96	1.04	Aldehyde
n-Hexadecanoic acid	C_16_H_32_O_2_	256	33.90	0.94	Fatty acid	1,3-Benzodioxol-5-ol	C_7_H_6_O_3_	138	19.32	0.24	Benzodioxole
Heptadecanoic acid, 9-methyl-, methyl ester	C_19_H_38_O_2_	298	38.58	0.44	Fatty acid ester	Methyl 4,4,7-trimethyl- 4,7-dihydroindan-6-carboxylate	C_14_H_20_O_2_	220	24.04	1.36	Indane
Hexadecanamide	C_16_H_33_NO	255	40.11	0.57	Fatty amide	E-7-Tetradecenol	C_14_H_28_O	212	27.59	0.11	Fatty alcohol
trans-13-Octadecenoic acid	C_18_H_34_O_2_	282	42.30	4.04	Fatty acid	7,9-Di-tert-butyl-1-ox aspiro(4,5) deca-6,9-d iene-2,8-dione	C_17_H_24_O_3_	276	32.98	0.53	Sspirocyclic diketone
9-Octadecenamide, (Z)-	C_18_H_35_NO	281	44.39	1.4	Fatty amide	trans-13-Octadecenoic acid	C_18_H_34_O_2_	282	34.29	15.39	Fatty acid
Eicosanoic acid, ethyl ester	C_22_H_44_O_2_	340	45.05	0.36	Fatty acid ester	9,12-Octadecadienoic acid, methyl ester, (E, E)-	C_19_H_34_O_2_	294	37.65	4.86	Fatty acid ester
Phenol, 2,2′-methylenebis[6-(1,1-dimethylethyl)-4-methyl-]	C_23_H_32_O_2_	340	45.64	1.94	Phenol	9-Octadecenoic acid, methyl ester, (E)-	C_19_H_36_O_2_	296	37.84	0.70	Fatty acid ester
Methyl erucate	C_23_H_44_O2	352	46.93	1.71	Fatty acid ester	13-Docosenamide, (Z)	C_22_H_43_NO	282	40.05	55.96	Fatty amide
Isochiapin B	C_19_H_22_O_6_	346	47.03	1.10	Sesquiterpene lactone	Octadecanoic acid	C_18_H_36_O_2_	284	40.5	3.74	Fatty acid
1,2-Benzenedicarboxylic acid	C_24_H_38_O_4_	390	47.96	0.83	Carboxylic acid	Phenol, 2,2′-methylenebis[6-(1,1-dimethylethyl)-4-methyl-]	C_23_H_32_O_2_	340	45.69	1.83	Phenol
13-Docosenamide, (Z)-	C_22_H_43_NO	337	51.86	78.41	Fatty amide	Linoleic acid ethyl ester	C_20_H_36_O_2_	308	46.59	0.81	Fatty acid ester
Ethyl iso-allocholate	C_26_H_44_O_5_	436	52.43	0.44	Steroid	12-Methyl-E, E-2,13 octadecadien-1-ol	C_19_H_36_O	280	47.06	0.17	Fatty alcohol
Oleic acid, 3-(octadecyloxy)prop yl ester	C_39_H_76_O_3_	592	54.88	2.89	Fatty acid ester	n-Propyl 9,12-octadecadienoate	C_21_H_38_O_2_	322	48.01	0.68	Fatty acid ester
Dotriacontane	C_32_H_66_	450	56.43	1.58	Alkane	Oleic Acid	C_18_H_34_O_2_	282	49.73	1.48	Fatty acid
						trans-9-Octadecenoic acid, pentyl ester	C_23_H_44_O_2_	352	50.45	3.74	Fatty acid ester
						Ethyl iso-allocholate	C_26_H_44_O_5_	436	51.73	1.03	Steroid
						n-Hexadecanoic acid	C_16_H_32_O_2_	256	52.43	0.76	Fatty acid
						9-Octadecenoic acid (Z)-, oxiranylmethyl ester	C_21_H_38_O_3_	338	54.27	0.95	Fatty acid ester
						Dotriacontane	C_32_H_66_	450	55.73	2.57	Alkane
						ç-Sitosterol	C_29_H_50_O	414	57.01	1.89	Phytosterol

**Table 2:** Anti-*H. pylori* activity, MIC, and MBC of the tested oil types. Data are presented as the mean ± SD. Different letters above numbers in the same column indicate a significant difference (*p* ≤ 0.05).

Sample Code	Inhibition Zone (mm)	MIC µg/mL	MBC µg/mL	MBC/MIC
Crude lettuce oil	13.7 ± 0.6 ^a^	62.5 ± 0.3 ^a^	125 ± 0.8 ^a^	2 ^a^
Lettuce oil + O_3_	21.3 ± 0.3 ^a^	31.25 ± 0.5 ^b^	62.5 ± 0.2 ^b^	2 ^a^
Standard drug	15.3 ± 0.4 ^a^	31.25 ± 0.6 ^b^	13.25 ± 0.6 ^c^	1 ^b^

**Table 3:** Docking scores and energy terms for (Z)-13-docosenamide and trans-13-octadecenoic acid docked into the crystal structure of human butyrylcholinesterase (BChE) (PDB: 4TPK).

Mol	S	rmsd_refine	E_conf	E_place	E_score1	E_refine	E_score2
13-Docosenamide	−8.13092	1.5926156	−19.6867	−72.8444	−9.60851	−39.1181	−8.13092
13-Docosenamide	−8.04835	1.7194235	−20.1995	−74.5038	−9.65519	−41.7513	−8.04835
13-Docosenamide	−7.62981	2.3539269	−19.1456	−40.5661	−10.4494	−41.0256	−7.62981
13-Docosenamide	−7.54724	1.1664486	−7.69996	−69.2003	−9.77358	−36.6556	−7.54724
13-Docosenamide	−7.52264	1.743559	−12.6639	−74.552	−9.41631	−32.9138	−7.52264
trans-13-Octadecenoic acid	−7.31024	2.458077	−13.897	−58.9793	−9.9934	−40.7344	−7.31024
trans-13-Octadecenoic acid	−7.19911	1.197924	−13.8079	−67.8339	−9.94211	−32.6767	−7.19911
trans-13-Octadecenoic acid	−7.19094	1.1504909	−16.0386	−58.7043	−9.75688	−39.1273	−7.19094
trans-13-Octadecenoic acid	−7.10033	3.2766879	−16.3373	−62.915	−10.9963	−36.7538	−7.10033
trans-13-Octadecenoic acid	−7.0948	1.0196184	−11.9226	−62.6675	−10.2318	−34.4483	−7.0948

**Table 4:** Interactions of (Z)-13-docosenamide and trans-13-octadecenoic acid with the structure of human butyrylcholinesterase (BChE) (PDB: 4TPK).

Mol	Ligand	Receptor	Interaction	Distance (Å)	E (kcal/mol)
13-Docosenamide	N 2	OE1 GLU 276 (A)	H-donor	3.42	−0.6
	C 35	5-ring TRP 82 (A)	H-pi	3.72	−0.7
trans-13-Octadecenoic acid	O 1	O SER 287 (A)	H-donor	2.97	−1.3
	C 39	6-ring TRP 82 (A)	H-pi	3.69	−0.8

**Table 5:** Docking scores and energy terms for (Z)-13-docosenamide and trans-13-octadecenoic acid docked into the crystal structure of *H. pylori* urease (PDB: 1E9Z).

Mol	S	rmsd_refine	E_conf	E_place	E_score1	E_refine	E_score2
13-Docosenamide	−6.00058	2.5410826	−21.2019	−42.7297	−9.22741	−32.8763	−6.00058
13-Docosenamide	−5.9666	2.395309	−19.3768	−88.8088	−9.60097	−32.495	−5.9666
13-Docosenamide	−5.9415	1.8721349	−17.2656	−71.2884	−9.55546	−31.9705	−5.9415
13-Docosenamide	−5.88933	2.118561	−21.9907	−57.6622	−9.80536	−31.4298	−5.88933
13-Docosenamide	−5.88896	2.0570378	−15.985	−69.9133	−8.96797	−29.6238	−5.88896
trans-13-Octadecenoic acid	−7.33015	1.6620129	−19.1551	−54.2166	−9.41694	−36.3137	−7.33015
trans-13-Octadecenoic acid	−7.15466	1.249053	−20.4189	−52.7527	−9.34551	−35.3725	−7.15466
trans-13-Octadecenoic acid	−7.02536	1.0522252	−13.2661	−42.4143	−9.99574	−26.2253	−7.02536
trans-13-Octadecenoic acid	−6.93917	1.3046346	−18.3703	−59.747	−9.90554	−33.8415	−6.93917
trans-13-Octadecenoic acid	−6.86541	2.0998428	−19.6161	−42.7127	−9.0778	−34.6881	−6.86541

**Table 6:** Interactions of (Z)-13-docosenamide and trans-13-octadecenoic acid with the structure of *H. pylori* urease (PDB: 1E9Z).

Mol	Ligand	Receptor	Interaction	Distance	E (kcal/mol)
13-Docosenamide, (z)	O 1	NH1 ARG 375 (B)	H-acceptor	3.05	−0.7
trans-13-Octadecenoic acid	O 1	OE1 GLU 313 (B)	H-donor	2.83	−6.4

## Data Availability

The original contributions presented in the study are included in the article, further inquiries can be directed to the corresponding author.
